# Development of a discrete choice experiment—an instrument to weight the preferences of registered nursing undergraduates to future employers: a descriptive study

**DOI:** 10.3389/frhs.2026.1804999

**Published:** 2026-05-01

**Authors:** Jana Bartakova, Martine Sophie Amrein, Diana Trutschel, Michael Simon

**Affiliations:** 1 Institute of Nursing Science, Department of Public Health, University of Basel, Basel, Switzerland; 2 Health Economics Facility, Department of Public Health, University of Basel, Basel, Switzerland; 3 Intensive Care Unit, University Hospital Basel, Basel, Switzerland

**Keywords:** discrete choice experiment (DCE), job attributes, job preferences, nurse workforce, switzeralnd, workforce retention

## Abstract

**Background:**

Healthcare systems worldwide, including in Switzerland, struggle to retain nurses and make the profession appealing to young people. Studies identify factors influencing Swiss nurses' job attraction and retention, but these factors are often presented as simple lists without insights into their impact—a crucial aspect for making informed policy decisions. This gap highlights the need for a strategy to assess these factors and their weight on nurses' career decisions.

**Aim:**

To develop and pretest a Discrete Choice Experiment (DCE) questionnaire that effectively measures the importance of various job attributes (i.e., job characteristics) influencing Swiss registered nurse graduates' employment decision, while also revealing their weight of influence.

**Design, methods:**

The development of the DCE questionary involved three phases: (1) Identifying attributes and assigning levels (e.g., leadership: yes/no) based on a literature review and interviews with experts; (2) Selecting the statistically optimal D-efficient experimental design and constructing choice sets; and (3) Developing the questionnaire and two pretesting with data collection, targeting graduating classes of registered nurses. The final DCE included nine attributes with two or three levels and consisted of 18 choice sets, each presenting three hypothetical jobs defined by different combinations of attribute levels, from which participants selected their preferred option. The survey also incorporated an internal consistency test and socio-demographic data collection. The two pretests included interviews with volunteer respondents.

**Results:**

The first pretest with 67 students (mean age 23.6 ± 2.8 years; 82.1% female) led to wording adjustments for four attributes and two levels. The second pretest with 53 students (mean age 25.3 ± 4.3 years; 67.9% female) confirmed the survey's clarity, lack of hypothetical job dominance, effective trade-off enforcement, and completion time of 15–30 min.

**Discussion:**

The second pretest revealed shifts in attribute prioritisation following questionnaire refinements, demonstrating that changes in attribute wording can meaningfully influence perceived importance and highlighting the critical role of precise definitions in shaping respondents' preferences.

## 1 Introduction

The global healthcare system is facing an acute nursing workforce crisis. Although widespread shortages of nurses were evident well before the COVID-19 pandemic, the pandemic further intensified these deficits, amplifying pressures on staffing capacity, care quality, and workforce sustainability (1, 2). Recent analyses indicate that global demand for nurses continues to outpace supply, with the World Health Organization estimating a shortfall of 4.5 million nurses by 2030 (3). Parallel evidence from peer-reviewed research highlights that the pandemic accelerated attrition, burnout, and mental health strain among nurses (4, 5). The compounded impact of chronic workforce shortages and post-pandemic strain has elevated the stabilization and expansion of the nursing workforce to a top priority on the global health policy agenda, essential for ensuring the resilience and sustainability of healthcare systems (1).

Within this broader context, early-career registered nurses (RNs) represent a particularly vulnerable group. Emerging evidence indicates that up to 40% of early-career RNs in South Africa leave their first positions within two years of practice (6), while in the United States, up to 33% exit the profession entirely within the same timeframe (7). In Switzerland, 18% of RNs reported an intention to leave the profession, with an additional 24% indicating that their decision would depend on future circumstances (8). Such early exits not only exacerbate staffing gaps but also reflect a generational shift in work motivations. Unlike past cohorts for whom nursing was often a lifelong vocation rooted in altruism (9), today's early-career RNs place greater emphasis on work–life balance, supportive leadership, professional growth opportunities, and fair compensation (10, 11). Evidence indicates that young nurses seek meaningful work and career satisfaction, but also insist on manageable schedules, adequate support, and career development prospects to stay in the profession (12, 13).

Better understanding which job characteristics early-career RNs value most, and how they trade off between them, is key to informing evidence-based strategies for nurse retention. Health economists and policymakers are increasingly turning to Discrete Choice Experiments (DCEs) to rigorously quantify such preferences. A DCE is a robust, utility-based method that presents individuals with hypothetical choice scenarios to reveal implicit preferences (e.g., salary, workload, scheduling, career advancement) and how they trade them off when making decisions (14, 15). Recently, this approach has gained prominence as a valuable tool for examining healthcare workers' job and employment preferences (16). By eliciting the relative importance of different job conditions, DCEs can inform targeted interventions—from compensation packages to workplace improvements—tailored to nurses' trade-offs and implicit preferences, that is, those revealed through actual choices rather than self-reported statements. Such evidence can support hospital managers and policymakers in designing retention strategies that align with the workforce's actual priorities and reveal meaningful trade-offs. This approach moves beyond compiling exhaustive lists of needs—often financially unrealistic or prone to overestimating actual priorities—and instead focuses on identifying the most impactful and feasible factors to enhance job satisfaction, reduce premature turnover, and sustain high-quality care delivery over the long term.

Despite widespread recognition of these issues, evidence remains limited regarding the implicit job preferences and trade-offs of early-career RNs in Switzerland, particularly in how they prioritize and balance key job characteristics in their employment decisions. Addressing this gap is essential for developing effective, context-specific policies to retain early-career RNs within the Swiss health system, while simultaneously identifying solutions that align with nurses' preferences and remain financially sustainable for healthcare management. This study aims to develop a DCE capable of effectively identifying early-career RNs' implicit job preferences and quantifying their trade-offs in Switzerland. By doing so, we seek to generate actionable insights into which improvements or incentives would most encourage new RNs to remain in the profession—knowledge that can directly inform workforce policy and help secure the future of Switzerland's nursing workforce.

## 2 Materials and methods

This paper reports on the development of a DCE, applying descriptive and exploratory approaches. The development of the DCE for the current research question was guided by the user guide „How to conduct a discrete choice experiment for health workforce recruitment and retention in remote and rural areas“ and papers about the construction of a DCE (17–22). The development process had three phases: 1) Identification of attributes and assignments of levels; 2) Selection of experimental design and construction of choice sets; 3) Development of the questionnaire and pretesting, including statistical analysis.

### 2.1 Phase 1: identification of attributes and assignments of levels

A literature search was undertaken to identify nurses' needs related to their careers and work environments, thereby informing the selection of job characteristics (i.e., attributes) relevant to the study population. To formulate a research question “What factors influence nurses in training in their choice of future professional setting/ job preferences?” and search strategy, we followed PICO framework (23). Guided by this framework, the population term “Students, Nursing*” and the outcome terms “Career Choice*” and “Choice Behavior*” were selected to further formulate the final search string (Supplementary Appendix 1). The Filter “publication date 10 years” as well as the requirement that the paper had to be published in English or German were added. Literature search was performed in April 2023 using PubMed/MEDLINE database and identified a total of 461 records. Based on an initial relevance assessment, 37 publications were selected for full-text review. In addition, backwards and forward snowballing of reference lists was conducted (24), resulting in the inclusion of 14 further relevant articles. These additional publications complemented the core literature and provided further contextual and conceptual insights relevant to the aims of the review.

From the comprehensive list of attributes identified through the literature search, snowballing, and document review, the research team preselected those most relevant to the Swiss context. To enhance content validity and incorporate system-level perspectives, guided expert panel interviews were conducted with ten professionals purposively sampled from a range of Swiss healthcare settings (rehabilitation, home care services, nursing education, hospital nursing services, psychiatric clinics, and paediatric care). In addition, three nursing students were interviewed to explore which conditions they considered important for remaining in the profession.

All interviews followed a semi-structured guide and were documented through detailed notes. The notes were analysed using an iterative, team-based qualitative synthesis. Members of the research team systematically reviewed and compared feedback across interviews, focusing on the relevance, clarity, feasibility, and policy actionability of candidate attributes and their potential levels. Particular attention was paid to identifying overlap and redundancy. Competing or overlapping attributes were initially discussed and, where possible, resolved already through guided consensus within the expert panels. Any remaining redundancies were subsequently addressed in structured research team discussions.

Attribute levels were defined to ensure both empirical realism and meaningful variation. Where applicable, levels were informed by current Swiss conditions and existing distributions to enhance credibility. At the same time, some of the selected levels intentionally reflected policy-relevant improvements beyond current practice (e.g., higher salary levels), particularly in areas where nurses and experts indicated a need for change. To support this process, the research team also consulted Swiss policy documents, publicly available reports and data sources, as well as descriptions of ongoing Swiss research and pilot initiatives aimed at improving working conditions. Overall, attributes and levels were derived from practice-based variation described by experts and iteratively refined to ensure interpretability, contextual validity, and policy relevance within the Swiss healthcare setting.

Insights from this process ultimately guided the selection of nine attributes and the determination of their corresponding levels, comprising six attributes with two levels and three with three levels.

### 2.2 Phase 2: selection of experimental design and construction of choice sets

The experimental design forms the methodological foundation of a DCE. It determines the structure and dimensionality of the choice experiment and applies statistical techniques to allocate attribute levels across choice tasks in an efficient and balanced manner (24). The number of all possible hypothetical scenarios in a DCE (so-called full factorial design) is determined by the number of attributes and their respective levels. In the case where attributes take on only two different numbers of attribute levels, the total number of hypothetical scenarios in a full factorial design equals a^n^ times b^m^, with a and b representing the respective number of attribute levels and *n* and m the number of attributes with those numbers of levels. In our case, six attributes with two levels and three attributes with three levels would lead to 1'728 hypothetical scenarios. Thus, to minimize respondent cognitive burden while maintaining statistical efficiency, a fractional factorial design was used.

The statistically optimal design used to select the most informative subset of hypothetical scenarios and construct the choice sets was a D-efficient design assuming zero priors for all parameters, as no prior parameter estimates were available. Consequently, a Bayesian D-efficient design based on empirical priors was not implemented. To balance D-efficiency—which decreases with an increasing number of attributes and levels but improves in designs with more alternatives—we included three job alternatives per choice set instead of only two (25). Generic alternatives (“Job A,” “Job B,” “Job C”) were used, as these labels carry no intrinsic meaning and therefore allow respondents to focus solely on the attribute levels rather than the job titles (26). A forced-choice format (i.e., no opt-out option) was applied to encourage respondents to make explicit trade-offs between attributes (27).

Following established methodological guidance, we selected a design comprising 18 choice sets, chosen so that the total number of sets is divisible by the number of levels for each attribute. This ensured attribute-level balance and further enhanced D-efficiency, serving as a design optimization strategy rather than a strict methodological requirement (27).

After constructing the choice sets, we performed several checks to assess key properties of the experimental design. First, a manual dominance check was conducted to identify whether any job alternative within the choice sets would be clearly preferred by the majority of respondents. Dominant alternatives do not provide meaningful information on respondents' trade-off behavior, as participants are likely to consistently select the clearly superior option (28). Consequently, these tasks contribute little to understanding the relative importance of attributes (29) and may distort scale parameters or bias estimated coefficients (30).

Second, we evaluated orthogonality and level balance of the final experimental design. Orthogonality was examined by computing pairwise Pearson correlations between the effect-coded design columns. Level balance was assessed by inspecting the frequencies and proportions of attribute levels across all profiles in the design. These diagnostics were used to verify that the design approximated orthogonality and that attribute levels were reasonably balanced, supporting reliable parameter estimation.

Theoretically, increasing the number of choice sets beyond 18 would provide additional information. However, the number of job choice tasks that respondents can reliably complete is limited, as cognitive burden increases with task complexity. Beyond a certain point, respondents may no longer be able to carefully evaluate and compare job alternatives (21). In health-economics DCEs, it is common for respondents to complete around 9–16 choice sets (31). Thus, to avoid increased respondent fatigue and the risk of dropout (22), the blocking procedure was applied. This approach divided the full 18-choice-set design into two sub-designs, each comprising nine randomly selected choice sets, while maintaining level balance. Each sub-design was intended for distribution to a distinct subset of students.

Following the first pretest of Phase 3 (described in subsection 2.3), the manual dominance check was replaced by an automated procedure implemented directly in the code generating the experimental design. The algorithm used two nested while loops: the inner loop searched for a D-efficient design, and once identified, the process advanced to the outer loop, which verified the design's balance. Specifically, we applied a simple constraint-based balancing rule based on the difference in total scores between jobs within each choice set, ensuring that no job exceeded another by more than 4.5 points. To calculate total scores, a scoring matrix was created in which each attribute level was assigned a value reflecting its relative attractiveness—from 0 points for the least attractive level to 1 point for the most attractive level within the same attribute (Table 1). This rule operationalises the idea of utility balancing to avoid dominant alternatives and extreme utility differences, as recommended in DCE design literature (30, 32). Whereas these approaches incorporate balance or non-dominance criteria directly into the D-efficient search algorithm, our procedure follows a simpler two-step approach: we first generate a standard D-efficient design and then apply a *post hoc* dominance check based on the point-scoring rule, discarding and regenerating designs that contain dominant alternatives. This approach avoids the complexity of fully constrained D-efficient or utility-balanced search algorithms while still enforcing the key requirement that choice sets do not contain dominant alternatives.

**Table 1:** Attributes, their levels and points—before and after pretests.

Attribute	Level A –0 point	Level B –0.5 point	Level C –1 point
Annual salary (brutto)	65`000 – 74`999 CHF	75`000 – 84`999 CHF	85`000 – 95`000 CHF
Time required for the journey to work (oneway, door-to-door, with preferred mode of transport)	60 Minutes		30 Minutes
Weekly working hours (100 % Workload)	42 Hours		38 Hours
Vacation days per year	20 Days		30 Days
How often do nursing actions have to be omitted?	Rarely		Frequently
How often do you work overtime or have to skip breaks?	Several times a week	Several times a month	Never
Influence on the shift plan	No	Yes, I can specify 5 desired shifts per month	Yes, I can write the entire shift plan myself
Influence on the shift plan (desired shift = desired time off, early, late or night shift)	Up to 3 desired shifts per month		At least 4 desired shifts up to writing the plan completely by myself
Standing in - additional shifts on originally free days	Yes, about once a week	Yes, about once a month	No
Supportive leadership - help with your career	No		Yes
Supportive leadership - individual promotion of strengths and professional development opportunities	No		Yes
Possibility to work additional night/weekend shifts in return for a financial bonus (CHF 25 per hour during the additional shifts worked)	No		Yes
Possibility to work additional night/weekend shifts in return for a financial bonus (CHF 25 per hour in addition to the regular salary during the additional shifts worked)	No		Yes

Attributes and levels shown in black represent the final version after two pretests; those in grey indicate the original attributes and levels that were modified during the pretesting phase. The assigned level points were applied in the search process to achieve a balanced design (see Section 2.2).

Additionally, a consistency test (Supplementary Appendix 3) was added to the design as the first choice set, identical for all participants. In this test, one job alternative clearly dominated the others, serving to assess whether respondents correctly understood the DCE task and decision logic (21).

### 2.3 Phase 3: development of the questionnaire and pretesting, including statistical analysis

The choice sets, along with the introductory text outlining the study's aim, purpose, and the role of nursing students in the pretest, as well as the sociodemographic questionnaire (Supplementary Appendix 2), were entered into the REDCap survey platform. To ensure data accuracy, a second researcher independently verified all entries for potential errors. The survey was then tested and validated by the data officer. Finally, a QR code was generated to facilitate convenient smartphone-based survey participation.

Two pretest studies were conducted by the research team at the Basel Center for Nursing Education, where the theoretical training of future registered nurses takes place. The first pretesting (groups 1 and 2) was carried out in September 2023, and the second (groups 3 and 4) in March and September 2024. The second pretest was conducted in two rounds due to a low participation rate in March. All registered nursing students who were in their final year of study at the time of pretesting (September 2023, March 2024, or September 2024) were invited to participate. A clarification of responsibility by the Ethics Committee of Northwest and Central Switzerland revealed no responsibility.

The pretesting was conducted in German and on students' official class time. Respondents were randomly divided into two groups to ensure balanced distribution across the two survey versions (with nine choice set subsets each). Randomization was implemented using a simple allocation procedure in which each participant drew a numbered chocolate bar and entered the corresponding number at the start of the survey. Immediately after the first pretest, follow-up interviews were conducted with volunteer participants. These interviews aimed to assess participants' understanding of the DCE concept, the feasibility of the number of choice sets, and the usability of the survey format. In addition, students were invited to provide feedback on the clarity and relevance of the presented attributes and levels and to indicate whether any required modification.

Between the two pretests, a group-guided interview was conducted in January 2024 with 18 final-year students from the Basel Center for Nursing Education, drawn from a different cohort than the participants in pretest 1. This phase aimed to ensure that the final modifications made after the first pretest were acceptable and meaningful to the target population before conducting the second pretest. During the session, students were presented with the revised version of the DCE and invited to collectively evaluate the selected attributes and levels. The discussion focused specifically on the comprehensibility, perceived realism, and relevance of each attribute and its levels in relation to students' anticipated transition into the Swiss labor market. Participants were encouraged to reflect on whether any attributes should be added, removed, or reformulated, and whether level ranges were considered realistic and appropriate.

Pretest data were exported from REDCap in CSV format to analytically validate the DCE questionnaire. Data were cleaned and checked for completeness, and only responses from participants who passed the internal consistency test were included in the analysis. A conditional logistic regression analysis was then performed to identify which attributes were most influential in job selection (17). Additionally, coefficient estimates for all attributes were calculated. Sociodemographic data were processed separately from the choice set data for each pretest. Therefore, means and standard deviations (SD), frequencies (n) and percentages (%) were calculated. To assess differences between samples, independent-samples t-tests were applied to continuous variables, and Pearson's Chi-square tests were used for categorical variables.

The experimental design and all analyses were conducted using R version 4.3.1 (“Beagle Scouts”). The package “idefix” (33) and “reshape2” (34) were used for data handling and formatting, “corrplot” (35) and “combinat” (36) for combination and permutation procedures, while the “survival” (37) and “ggplot2” (38) packages were applied for regression analysis and data visualization, respectively.

## 3 Result

In the first pretest, 67 graduating nurses participated, and 21 of them took part in follow-up interviews. The second pretest included 53 graduating nurses (34 in March and 19 in September), with 17 (11 in March and 6 in September) participating in subsequent interviews. One participant from the first pretest was excluded due to incomplete responses. All remaining participants passed the internal consistency test and were therefore included in the statistical analysis. Based on school administration data indicating that each graduating cohort comprised approximately 90 students, both pretests achieved response rates exceeding 58%.

Across both pretests, the majority of participants were female, of Swiss nationality, and intended to remain near their hometown after graduation. Most were single, without children, and not responsible for caregiving. The first and second pretest samples differed slightly with a significant difference in age and in whether participants had completed prior vocational training as licensed practice nurses. There were no significant differences in gender distribution or educational background, and both samples showed similar proportions preferring employment in acute hospital settings and expressing interest in clinical qualification (see Table 2 for details). Differences between the March and September 2024 rounds of the second pretest were examined. As they exhibited the same pattern of differences as observed between the first pretest (September 2023) and the second pretest overall (March and September 2024), the results for March and September 2024 are not presented separately.

**Table 2:** Participants' characteristics—pretest 1 and 2.

Characteristic	Pretest 1 (group 1, 2)	Pretest 2 (group 3, 4)
	September 2023	March, September 2024
Participants	67 (100%)	34 + 19 = 53 (100%)
Sociodemographic variables		
Age (years)^c^	23.6 ± 2.8	25.3 ± 4.3
Female	55 (82.1%)	36 (67.9%)
Swiss Nationality	57 (85.1%)	41 (77.4%)
School education		
Compulsory school	38 (57.6%)	30 (56.6%)
Specialized Maturity Diploma	14 (21.2%)	11 (20.8%)
Maturity Diploma	14 (21.2%)	12 (2.6%)
NA	1 (1.5%)	
Prior Licensed Practical Nurse Qualification^c^	53 (79.1%)	31 (58.5%)
Marital status		
Single	42 (62.6%)	36 (67.9%)
Engaged	19 (28.4%)	9 (17.0%)
Married	5 (7.5%)	7 (13.2%)
Divored	1 (1.5%)	1 (1.9%)
Living with parents	35 (52.2%)	28 (52.8%)
NA		1 (1.9%)
Parent's education		
(Applied) University	16 (23.9%)	13 (24.5%)
Vocational Education (Higher Technical College)	21 (31.3%)	22 (41.5%)
Vocational Training (Federal Certificate of Competence)	21 (31.3%)	13 (24.5%)
Compulsory school	5 (7.5%)	3 (5.6%)
Other	4 (6.0%)	1 (1.8%)
I don`t know	0 (0.0%)	1 (1.8%)
Care about grandparents, chronically ill parent/sibling	18 (26.9%)	13 (24.5%)
Having children	4 (6.0%)	4 (7.5%)
Importancy to stay near hometown (0 = not, 100 = very)	70.4 ± 21.9	69.0 ± 23.7
NA	6 (9.0%)	9 (17.0%)
Preferred setting of work after studies^b^		
Acute care/Hospital	50 (74.6%)	39 (73.6%)
Psychiatry	13 (19.4%)	13 (24.5%)
Rehabilitation	4 (6.0%)	10 (18.9%)
Nursing home	7 (10.5%)	6 (11.3%)
Home care	3 (4.5%)	7 (13.2%)
Paediatrics	14 (20.9%)	6 (11.3%)
Hospice/Palliative care	5 (7.5%)	4 (7.5%)
I don't want to continue working in nursing	13 (19.4%)	3 (5.6%)
Clinical qualification^a^	31 (46.3%)	27 (50.9%)
Management	11 (16.4%)	11 (20.8%)
Education (vocational training in practice or at school)	36 (53.7%)	22 (41.5%)
Academic (teaching/research at the university), PhD	4 (6.0%)	8 (15.1%)
I just want to work as a normal nurse at the moment	20 (29.9%)	14 (26.4%)
I don't know yet	8 (11.9%)	4 (7.5%)
Something else	9 (13.4%)	6 (11.3%)

^a^ such as Postgraduate studies/ Further Specialization in Intensive Care, Emergency, Anaesthesia/Certificate of Advanced Studies, Diploma of Advanced Studies, Master of Advanced Studies or as an Advanced Practical Nurse (Clinical Nurse Specialist/Nurse Practitioner).

^b^ Respondents were allowed to select multiple preferred work settings.

^c^ *t*-test/Chi^2^-test, *p* < 0.05.

Categorical variables are presented as counts (percentages). Continuous variables are given by mean ± standard deviation. Observations coded as NA are omitted from the calculation of group differences.

SD, Standard Deviation; NA, not available.

During the first pretest and group-guided interview in January 2024, students noted that most attributes reflected nurse-related dimensions, while an attribute describing workload by “How often do nursing actions have to be omitted?” shifted focus to the patient dimension. They perceived this as problematic, as it implicitly opposed nurse well-being to patient well-being. Given that individuals entering the nursing profession are often motivated to support patient care, this framing risked creating a moral conflict within the choice task. To address this issue, the workload attribute was revised for the second pretest to “How often do you work overtime or have to skip breaks?”, thereby maintaining the focus on nurse-centered working conditions. Additionally, the response levels for this attribute were expanded from a two-level structure (“rarely” and “frequently”) to a more nuanced three-level format: “several times a week,” “several times a month,” and “never” (Table 1: final attributes and levels in black; original attributes and levels that were modified during pretesting shown in grey).

Conversely, based on findings from the first pretest, the attribute “Influence on the shift plan” was reduced from three to two levels. As minimal difference was observed between the responses “Yes, I can specify five desired shifts per month” and “Yes, I can write the entire shift plan myself,” (Table 1) the revised levels were defined as “Up to three desired shifts per month” and “At least four desired shifts, up to writing the plan independently”, while the response option “No” was excluded. The wording of the other two attributes was identified as suboptimal during the first pretest and their wording was also refined based on the follow-up interview (see Table 1 for detailed revisions). During the second pretest and follow-up interview, no issues were reported.

Overall, participants found the survey process feasible, understandable, and manageable, typically completing it within 15–30 min, depending on individual pace.

**Figure 1:**
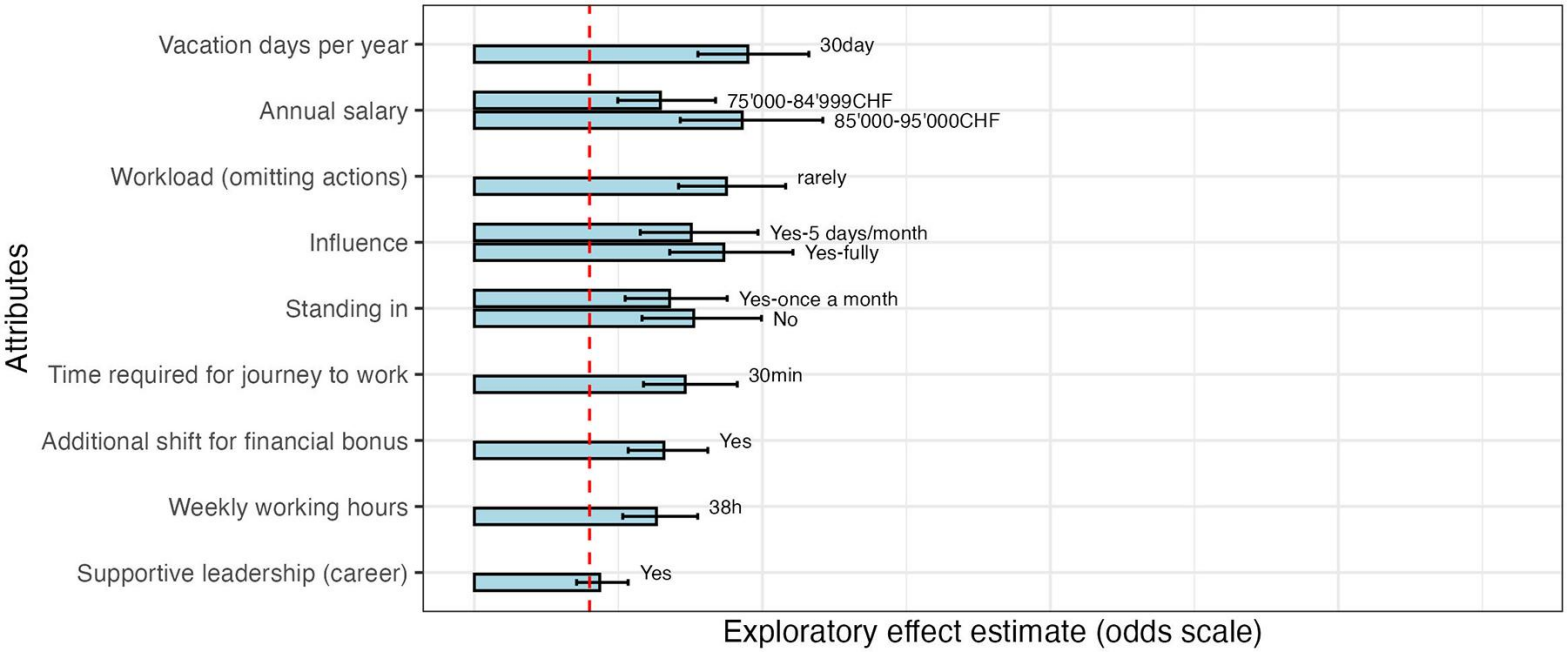
Exploratory attributes effects—odds-scale (pretest 1: group 1, 2). “Workload (omitting actions)” refers to “How often do nursing actions have to be omitted?”; “Influence” to “Influence on the shift plan”; “Standing in” to “Standing in—additional shifts on originally free days”; “Additional shift for financial bonus” to “Possibility to work additional night/weekend shifts in return for a financial bonus (CHF 25 per hour during additional shifts worked)”; and “Supportive leadership (career)” to “Supportive leadership—help with your career” (see Table 1).

**Figure 2:**
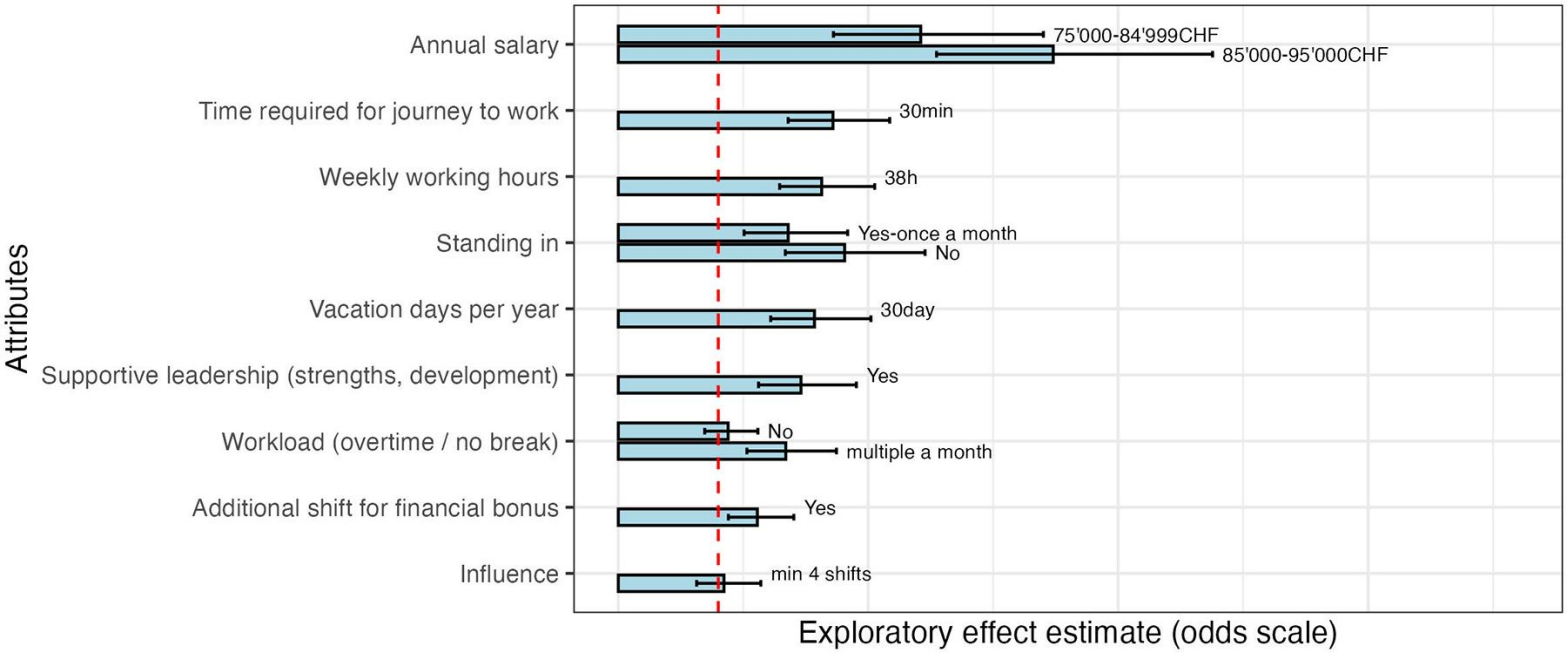
Exploratory attributes effects—Odds-scale (pretest 2: group 3, 4). “Standing in” refers to “Standing in—additional shifts on originally free days”; “Supportive leadership (strengths, development)” refers to “Supportive leadership—individual promotion of strengths and professional development opportunities”; “Workload (overtime/no break)” refers to “How often do you work overtime or have to skip breaks?”; “Additional shift for financial bonus” refers to “Possibility to work additional night/weekend shifts in return for a financial bonus (CHF 25 per hour in addition to the regular salary during the additional shifts worked)”; “Influence” refers to “Influence on the shift plan (desired shift = desired time off, early, late or night shift)” (see Table 1).

Figures 1, 2 present the results of statistical analysis from the first and second pretests, respectively. These analyses were conducted to assess the performance of the experimental design and the internal consistency of responses during instrument development. Given the small and non-representative pretest samples, the estimated odds ratios (ORs) are not intended to provide substantive evidence on preference strength or attribute ranking. Instead, they were used to confirm that attribute levels operated in the expected direction, that no dominant alternatives were present, and that respondents engaged in meaningful trade-offs.

In the first pretest (Figure 1), variation in parameter estimates across attributes indicated that respondents were able to differentiate between levels. Attributes with comparatively larger effect sizes suggested clearer discrimination between levels, whereas smaller effects signaled areas where wording or level specification required further refinement.

Following revisions to attribute phrasing and level definitions, the second pretest (Figure 2) demonstrated shifts in parameter estimates. These changes reflect the impact of revised wording and structural adjustments rather than substantive changes in underlying preferences. For example, refining the operationalization of workload—from the “frequency with which nursing activities must be omitted” to the “frequency of overtime work or skipped breaks”—and of supportive leadership—from “help with your career” to “individual promotion of strengths and professional development”—was accompanied by changes in parameter magnitudes. These shifts underscore the sensitivity of respondents' choices to precise attribute definitions and the importance of careful wording during instrument development. Due to adjustments in level structure, direct comparison for some attributes was not feasible. Overall, the second pretest indicated improved clarity, balance, and functional performance of the instrument prior to the main data collection.

## 4 Discussion

Pretesting demonstrated that the overall structure and content of the DCE questionnaire were feasible for the nursing student population. Once revisions were implemented based on the first pretest, all students participating in the second pretest reported that they could complete the choice tasks without difficulty. Furthermore, the use of QR-code access—which enabled completion on smartphones—proved to be convenient and well aligned with students' everyday digital habits. This low-threshold, user-friendly mode of delivery appears suitable for wider implementation and can be confidently adopted for the nationwide DCE survey across Switzerland.

The number of participants in the pretest studies was determined by attendance in the final class session of the nursing course at the Basel Center for Nursing Education. Although data collection was intentionally scheduled during official class time to maximize participation, attendance on the final course day was lower than anticipated—particularly during the March 2024 pretest. Therefore, an additional pretest was conducted in September 2024 using the same DCE questionnaire and procedures as in March. This approach ensured a sufficient number of respondents for the second pretest, with the variation in participants' characteristics between the March and September 2024 rounds mirroring the pattern observed between the first pretest (September 2023) and the combined second pretest. While the cognitive interviews in the first pretest yielded essential insights that informed the refinement of the instrument, the interviews conducted during the second pretest confirmed that the revised questionnaire was well understood and revealed no further concerns.

Refinements to the questionnaire after the first pretest led to a notable shift in the prioritisation of attributes. Given the exploratory and non-representative nature of the pretest samples, these shifts should not be interpreted as substantive changes in preference strength or attribute importance. Rather, they illustrate the sensitivity of respondents' choices to wording and level specification and underscore the central role of precise, clearly defined attributes in DCE development. Furthermore, misspecification of attributes or attribute levels can have substantial negative consequences for both the design and interpretation of a DCE, potentially leading to misleading preference estimates and reduced applicability of results in workforce planning (39). Such findings align with established methodological guidance emphasising that careful attribute development and clear conceptualisation are essential to the validity of stated preference research (40). This also underscores a broader limitation of relying solely on attributes derived from prior literature: without further contextualisation and adjustment. Published attributes may insufficiently reflect the perspectives, experiences, and decision processes of the actual target population (40). For example, we reviewed existing DCE studies that investigated job preferences among nursing students—including those conducted in Australia, China, Thailand, and Zambia (41–45). However, many of the attributes identified in these international studies were not directly transferable to the Swiss context. For instance, “Bianzhi”, a key component of the Chinese employment system in which staffing positions are allocated by the government, has no functional equivalent in Switzerland (41, 46). Similarly, the strong urban–rural divide observed in Zambia carries far less weight in Switzerland, given its highly developed mobility infrastructure (45). Other attributes commonly included in studies from low- and middle-income countries were not deemed relevant for Switzerland. Housing provision, for instance, plays an important role in Zambia and Thailand (43, 45), whereas in Switzerland it has lost significance over time. Although “nurses' homes” existed historically, they are now rare and hold little relevance in modern workforce planning. Likewise, attributes capturing availability of essential medical equipment—used in Zambia and China (41, 45)—were not included because Swiss healthcare facilities generally meet high infrastructural and supply standards. While temporary shortages of medicines or materials may occur due to global supply-chain issues, timely access to necessary medical interventions remains consistently ensured.

The annual gross salary range used in the DCE (85,000–95,000 CHF) may appear high at first glance, and it is reasonable to question whether the upper level exceeds what is typically achievable in the Swiss nursing labour market. However, when comparing median earnings of nurses with those of other higher-educated professionals, international evidence suggests that the relative earnings advantage associated with nursing and related health professions is below that of many other tertiary-educated fields, indicating that nurses can earn comparatively less despite similar education levels (47). For this reason, and in line with emerging calls to improve the competitiveness of nursing salaries, the study team deliberately included a higher wage level to capture students' preferences under realistic future scenarios rather than restricting the range to current median values. Within this context, the observed increase in the relative importance of salary between the two pretests should nevertheless be interpreted cautiously. Given the small and non-representative pretest samples, part of this shift may reflect sampling variability rather than substantive changes in underlying preferences. At the same time, the consistent prominence of remuneration across both rounds aligns with previous DCE evidence identifying salary effects underscores its central role in job decision-making, consistent with previous DCE studies that identified remuneration as a central determinant of job preferences among prospective and early-career nurses (41–44). Thus, while effect sizes in pretesting should not be overinterpreted, the overall pattern reinforces the importance of financial incentives in workforce decision-making.

The remaining continuous variables—time required for the journey to work, weekly working hours, and vacation days per year—were selected to reflect meaningful and realistic variation. All three are already subject to experimentation or policy debate in Switzerland. For instance, some hospitals have introduced 38-hour work weeks (48), while others have expanded annual leave entitlements up to 32 days (49). These developments underscore the relevance of including such attributes in a preference study aimed at informing workforce strategies.

An additional insight emerged from the qualitative interviews conducted in September 2023: many students reported that they could not imagine being responsible for drafting their own shift schedules. This finding is noteworthy, particularly because participatory scheduling models have been successfully implemented in several Swiss institutions. It may reflect limited exposure to participatory scheduling during training, assumptions about managerial authority, or life-stage factors—such as the absence of caregiving or family responsibilities—that shape how scheduling flexibility is evaluated. Interestingly, in the first pretest the absence of any influence over shift planning was associated with a clear behavioural effect, whereas in the second pretest this effect was reduced. This change likely relates to the revised operationalisation of the attribute, including the removal of the “no influence” level, rather than indicating that scheduling autonomy is unimportant. The comparison between pretests therefore underscores the sensitivity of categorical attributes to level specification and wording.

More broadly, defining categorical variables proved considerably more challenging than specifying continuous ones. Several refinements were therefore made after the first pretest. As Ryan et al. note, categorical attributes often require descriptive phrasing and are inherently susceptible to varied personal interpretation, making them more difficult to communicate uniformly across respondents (21). The most substantial modification involved replacing the attribute “How often do nursing actions have to be omitted?” with “How often do you work overtime or have to skip breaks?”. The intention was to capture subjective workload burden as objectively as possible—a concept also explored in Chinese DCEs through measures of workload pressure (41, 46) and in the Australian DCE using staffing-level indicators (42). The reduced effect observed after revision should therefore not be interpreted as evidence that workload is unimportant; rather, it likely reflects differences in framing and level definition, combined with the limited statistical precision inherent in small pretest samples.

We were also unable to observe a clear pattern in the pretest analyses consistent with prior studies suggesting that supportive leadership is a key determinant of job preference among young nurses. Previous research has highlighted that new graduates seek environments that foster development and where leadership demonstrates a supportive attitude (42, 50, 51). However, given the exploratory nature of the pretest samples, these findings should not be interpreted as evidence contradicting the existing literature. Rather, they may reflect sensitivity to attribute wording, limited statistical precision in pretesting, or the fact that students—who have had limited exposure to workplace hierarchies—may not yet fully appreciate the role of leadership in shaping professional satisfaction. The main study with early-career nurses will allow a more robust assessment of this attribute.

These findings collectively highlight the broader methodological challenge inherent in DCE development: ensuring that the selected attributes are both comprehensive and cognitively manageable. DCE methodology assumes that respondents consider all presented attributes and make conscious trade-offs among them. These trade-offs form the basis for estimating relative preferences and, ultimately, monetary values. However, if too many attributes or levels are included, participants may experience cognitive overload and resort to simplifying strategies—such as consistently choosing the option with the highest salary—which would undermine the validity of the estimated trade-offs (21). This raises the methodological question of how many attributes are appropriate. Existing DCE applications in health economics have used between two and 24 attributes, with six being the most common (15). The DCEs we drew upon as methodological references typically included five to seven attributes (41, 43–45). Although our inclusion of nine attributes places the instrument at the upper end of this range, it remains within established precedent, as demonstrated by the Australian DCE including 12 attributes (42). The balance between conceptual completeness and cognitive burden was therefore carefully considered throughout the design process.

Beyond the number of attributes, the internal balance of alternatives within each choice set is an important consideration in DCE design. Dominant alternatives—profiles in which all attribute levels are more desirable than those of competing options—are unlikely to generate meaningful trade-offs, as respondents can select them without evaluating attribute differences (28, 29). Such dominance reduces the informational value of responses and may influence scale and parameter estimation (30). Although avoiding dominance is widely recommended in the DCE literature, practical guidance on how to operationalise this during experimental design remains heterogeneous. Common approaches include manually removing dominant choice tasks after design generation (52) or specifying constraints during the design search, for example through the use of priors (29). However, these strategies may reduce D-efficiency or introduce correlation and imbalance across attribute levels (27, 53). More formal approaches incorporate utility balance or dominance restrictions directly within the design algorithm (30, 32), but these procedures are computationally more demanding and are not routinely implemented in applied DCE studies. Our approach represents a pragmatic implementation of the utility-balancing principle, using a two-step procedure: a standard D-efficient design search was conducted first, followed by a check for excessive differences in overall attractiveness between alternatives, thereby preserving both D-efficiency and utility balance. Although the concept of balancing expected utility across alternatives is well established in the DCE literature (30, 32), practical implementations vary widely. Future research could compare this type of *post hoc* constraint approach with fully integrated utility-balanced or dominance-restricted design algorithms in terms of design efficiency, respondent behaviour, and parameter stability.

In the wake of the COVID-19 pandemic, evidence shows that staffing shortages have intensified, contributing to ongoing workforce crises with implications for patient safety and service delivery (54, 55). Addressing this crisis cannot rely solely on training more nurses, particularly when resignation rates across age groups approach 40% (56, 57). This situation underscores the necessity of research aimed at understanding job preferences and retention factors, as well as the urgency with which such evidence must be translated into policy and organisational action. Notably, most young nurses in Switzerland do not intend to leave the profession; rather, they consistently express a desire for improved working conditions (51). This highlights the critical importance of identifying and implementing workplace attributes that can effectively enhance retention.

### 4.1 Strenght and limitations

This study contributes to addressing an important methodological gap: the need for transparent and detailed reporting of the qualitative and developmental processes used to derive DCE attributes and levels. Such information is essential for enabling readers to appraise the rigour and validity of the resulting choice experiment, yet it remains insufficiently documented in much of the existing literature across both high- and low-income settings (58, 59). By providing step-by-step insight into our attribute development and refinement procedures, this article offers practical guidance that may support future DCE design in health workforce research.

Nonetheless, several limitations must be acknowledged. A key limitation concerns the formative qualitative work informing attribute and level selection. Because the DCE targets nursing students, interviewing only three students in Phase 1 may have constrained our ability to capture heterogeneity in students' job expectations and to verify that the final attributes reflect the most influential decision drivers at the point of transition into practice. However, this was explicitly mitigated by additional validation steps: between the two pretests, we conducted a structured group interview with 18 final-year nursing students. In that session, students reviewed the revised DCE and collectively evaluated each attribute and its levels, reflecting on clarity, realism, and relevance in the context of their future careers. They indicated whether any attributes should be added, removed, or reformulated and whether level ranges were plausible. This group-based feedback broadened our sample of student voices and further informed refinements.

Importantly, while qualitative engagement with end users is widely regarded as good practice, the DCE literature provides limited operational guidance on minimum qualitative sample sizes, and reporting of qualitative attribute-development procedures remains inconsistent across healthcare DCEs (40, 60, 61). This creates a pragmatic tension between feasibility and methodological ideal, particularly in workforce-focused DCEs intended to inform policy (26). In our case, the small number of student interviews in phase 1 is balanced by deliberate follow-up validation and extensive stakeholder triangulation. In sum, while the limited initial sample is a constraint, it was mitigated by both rigorous expert input and additional student validation, leaving this as a methodological limitation rather than a fatal flaw.

The second methodological consideration concerns sample size requirements for DCEs. Although blocking is an established strategy to reduce cognitive burden, reliable estimation of preference parameters across blocks generally requires larger samples than were available in our pretests. In the second pretest, each of the nine choice sets per block was completed by 21–22 participants. While this is sufficient for assessing comprehension, logical consistency, and basic parameter directionality, it is not adequate for drawing substantive conclusions about preference strength or attribute importance.

The primary aim of both pretests was therefore methodological: to evaluate feasibility, test the experimental design, assess internal consistency, and refine attribute wording and level specification. Although 67 students participated in the first pretest and 53 in the second, these samples were not intended to provide statistically robust or generalisable preference estimates. Sample size considerations in DCE research are inherently linked to design complexity, number of parameters, and blocking structure; consequently, substantially larger samples are required for confirmatory analyses (31).

Accordingly, the pretest findings should be interpreted exclusively as part of the instrument development process. The main nationwide study will require a substantially larger and more diverse sample to enable reliable estimation of preference parameters and subgroup analyses.

Although this study does not provide representative evidence for Switzerland, it fulfills its intended purpose: the systematic refinement and methodological validation of the DCE instrument. The data were generated through pretesting with students from one institution, and no policy-relevant conclusions should be drawn from these preliminary analyses.

## Data Availability

The raw data supporting the conclusions of this article will be made available by the authors, without undue reservation.
