# Supplementary material for: Development of a discrete choice experiment—an instrument to weight the preferences of registered nursing undergraduates to future employers: a descriptive study
Source: Front Health Serv. 2026 May 1;6:1804999. doi: 10.3389/frhs.2026.1804999 (PMC13199924; doi:10.3389/frhs.2026.1804999)
Supplement: Supplementary file 1 [file Supplementaryfile1.docx]

**Appendix 1– Search String for PubMed**

| Block | MeSH Terms  Free Text (Title/Abstract)Search String | Hits |
| --- | --- | --- |
| Block P | Students, Nursing[MeSH Terms]  Students, Nursing[Title/Abstract]  Student, Nursing[Title/Abstract]  Pupil Nurse*[Title/Abstract]  Nurses, Pupil[Title/Abstract]  Nurse, Pupil[Title/Abstract]  Nursing Student*[Title/Abstract]  Nursing Undergraduate*[Title/Abstract]  "students, nursing"[MeSH Terms] OR "students nursing"[Title/Abstract] OR "student nursing"[Title/Abstract] OR "pupil nurse*"[Title/Abstract] OR (("nurse s"[All Fields] OR "nurses"[MeSH Terms] OR "nurses"[All Fields] OR "Nurse"[All Fields] OR "nurses s"[All Fields]) AND "Pupil"[Title/Abstract]) OR "nurse pupil"[Title/Abstract] OR "nursing student*"[Title/Abstract] OR "nursing undergraduate*"[Title/Abstract] | 30,103  334  72  40  148  3  20,370  452  37,114 |
| Block O | Choice Behavior[Mesh:NoExp]  Choice Behavior*[Title/Abstract]  Choice Behaviour[Title/Abstract]  Behavior, Choice[Title/Abstract]  Behaviors, Choice[Title/Abstract]  Approach Behavior*[Title/Abstract]  Approach Behaviour*[Title/Abstract]  Behavior, Approach[Title/Abstract]  Behaviors, Approach[Title/Abstract]  Behaviour, Approach[Title/Abstract]  Career Choice[MeSH Terms]  Career Choice*[Title/Abstract]  Choice, Career[Title/Abstract]  Choices, Career[Title/Abstract]  Discrete Choice Experiment[Title/Abstract]  Choice Nursing Discipline[Title/Abstract]  Working Condition*[MeSH Terms]  (working condition*[Title/Abstract])  "choice behavior"[MeSH Terms] OR "choice behavior*"[Title/Abstract] OR "choice behaviour"[Title/Abstract] OR "behavior choice"[Title/Abstract] OR "behaviors choice"[Title/Abstract] OR "approach behavior*"[Title/Abstract] OR "approach behaviour*"[Title/Abstract] OR "behavior approach"[Title/Abstract] OR "behaviors approach"[Title/Abstract] OR "behaviour approach"[Title/Abstract] OR "career choice"[MeSH Terms] OR "career choice*"[Title/Abstract] OR "choice career"[Title/Abstract] OR "choices career"[Title/Abstract] OR "discrete choice experiment"[Title/Abstract] OR (("choice behavior"[MeSH Terms] OR ("Choice"[All Fields] AND "Behavior"[All Fields]) OR "choice behavior"[All Fields] OR "Choice"[All Fields] OR "Choices"[All Fields]) AND "nursing discipline"[Title/Abstract]) OR "working condition*"[MeSH Terms] OR "working condition*"[Title/Abstract] | 34,843  1,732  352  54  3  786  167  89  20  41  25,368  3,531  21  5  2,421  7  118  6,868  78,903 |
| Block P and O | ("students, nursing"[MeSH Terms] OR "students nursing"[Title/Abstract] OR "student nursing"[Title/Abstract] OR "pupil nurse*"[Title/Abstract] OR (("nurse s"[All Fields] OR "nurses"[MeSH Terms] OR "nurses"[All Fields] OR "Nurse"[All Fields] OR "nurses s"[All Fields]) AND "Pupil"[Title/Abstract]) OR "nurse pupil"[Title/Abstract] OR "nursing student*"[Title/Abstract] OR "nursing undergraduate*"[Title/Abstract]) AND ("choice behavior"[MeSH Terms] OR "choice behavior*"[Title/Abstract] OR "choice behaviour"[Title/Abstract] OR "behavior choice"[Title/Abstract] OR "behaviors choice"[Title/Abstract] OR "approach behavior*"[Title/Abstract] OR "approach behaviour*"[Title/Abstract] OR "behavior approach"[Title/Abstract] OR "behaviors approach"[Title/Abstract] OR "behaviour approach"[Title/Abstract] OR "career choice"[MeSH Terms] OR "career choice*"[Title/Abstract] OR "choice career"[Title/Abstract] OR "choices career"[Title/Abstract] OR "discrete choice experiment"[Title/Abstract] OR (("choice behavior"[MeSH Terms] OR ("Choice"[All Fields] AND "Behavior"[All Fields]) OR "choice behavior"[All Fields] OR "Choice"[All Fields] OR "Choices"[All Fields]) AND "nursing discipline"[Title/Abstract])) | 1,510  Added filter “publication date 10 years”:  461 results left |

*Note:* Final Search was conducted on 14.04.2023
