# Supplementary material for: Development of a discrete choice experiment—an instrument to weight the preferences of registered nursing undergraduates to future employers: a descriptive study
Source: Front Health Serv. 2026 May 1;6:1804999. doi: 10.3389/frhs.2026.1804999 (PMC13199924; doi:10.3389/frhs.2026.1804999)
Supplement: Supplementary file 2 [file Supplementaryfile2.docx]

**Appendix 2 – Introduction DCE**

Hallo zukünftige Pflegefachperson

Euer heutiger wertvoller Beitrag kann die Verbesserung der Arbeitsbedingungen für Pflegefachpersonen massgeblich beeinflussen. Als aktives Mitglied des Schweizerischen Berufsverbands der Pflegefachfrauen und -männer engagiere ich mich aktiv für bessere Arbeitsbedingungen und habe dieses Thema auch für meine Masterarbeit gewählt. Dieses Projekt zielt darauf ab, die positive Veränderungen im Pflegebereich voranzutreiben. Zu diesem Zweck haben wir ein Discrete Choice Experiment (DCE) entwickelt, um herauszufinden, welche Aspekte eines Pflegeberufs ihn für Euch attraktiver machen. Eure Aufgabe dabei ist es, in den verschiedenen Szenarien das jeweils attraktivere Stellenangebot auszuwählen.

Die Umfrage richtet sich an angehende Pflegefachpersonen an Höheren Fachschulen und Fachhochschulen in der Deutschschweiz. Das DCE befindet sich derzeit in der Pilotphase und wird Euch vorgestellt, um im Anschluss noch einmal angepasst werden zu können. Die Teilnahme ist freiwillig, und Ihr könnt jederzeit aussteigen. Diese Umfrage ist ein erster wichtiger Schritt, um aufzuzeigen, wie der Pflegeberuf für die Zukunft attraktiver gestaltet werden kann. Euer Feedback zu den Berufsszenarien und deren Verständlichkeit ist sehr wertvoll.

Der DCE-Fragebogen besteht aus zwei Teilen:

1) Über Euch

2) diverse Szenario mit Jobbeschreibungen

Insgesamt sollte das Ausfüllen des Fragebogens etwa 15 bis 25 Minuten in Anspruch nehmen.

Wir versichern Euch, dass alle Angaben im Fragebogen streng vertraulich behandelt werden und Eure Daten sicher auf einem Server der Universität Basel gespeichert werden. Zusätzlich ist die Datenverarbeitung anonym und wird getrennt von der Angabe der Mailadresse verwendet.

Dankeschön für eure wertvolle Mitarbeit!
