# Supplementary figures and images for: Development of a discrete choice experiment—an instrument to weight the preferences of registered nursing undergraduates to future employers: a descriptive study
Source: Front Health Serv. 2026 May 1;6:1804999. doi: 10.3389/frhs.2026.1804999 (PMC13199924; doi:10.3389/frhs.2026.1804999)

**Appendix 3 – Example of the DCE choice set (internal consistency test of group 3, Pretest 2)**

*
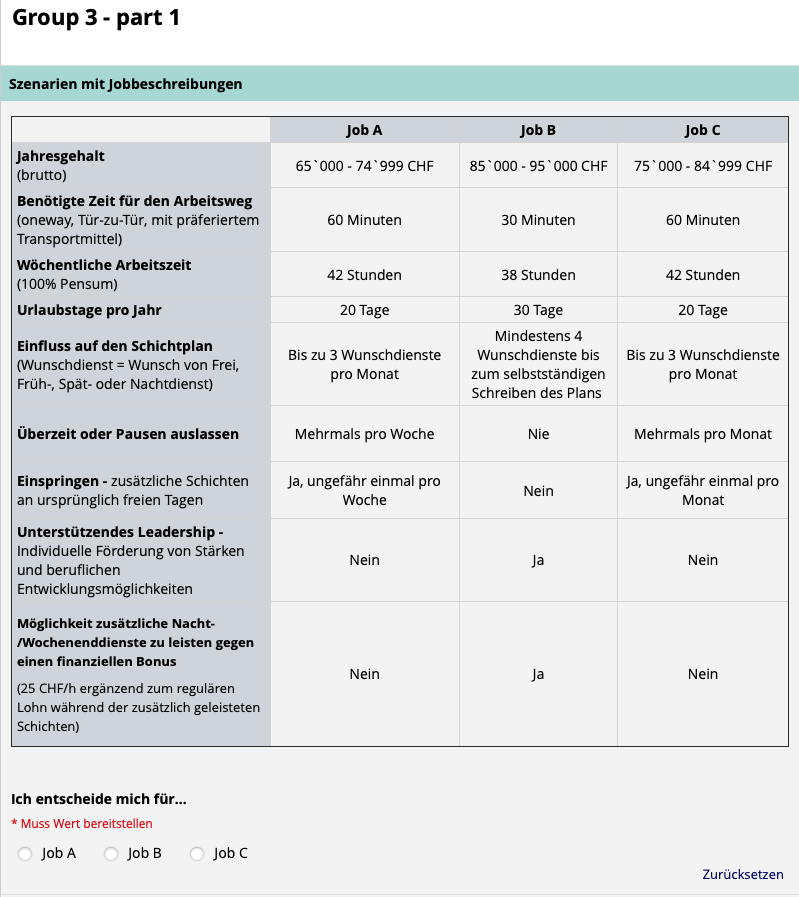
*

Supplement: Supplementary file 3 [file Supplementaryfile3.docx]
